# Nutrition Care after Hospital Discharge in Singapore: Evidence-Based Best-Practice Recommendations

**DOI:** 10.3390/nu15214492

**Published:** 2023-10-24

**Authors:** Doris Hui Lan Ng, Frederick Hong Xiang Koh, Hazel Ee Ling Yeong, Terence Cheong Wei Huey, Koy Min Chue, Fung Joon Foo, Samuel Teong Huang Chew

**Affiliations:** 1 Department of Gastroenterology and Hepatology, Tan Tock Seng Hospital, 11 Jalan Tan Tock Seng, Singapore 308433, Singapore; doris_hl_ng@ttsh.com.sg; 2 Department of General Surgery, Sengkang General Hospital, 110 Sengkang East Way, Singapore 544886, Singapore; frederick.koh.h.x@singhealth.com.sg (F.H.X.K.); chue.koy.min@singhealth.com.sg (K.M.C.); 3 Department of Nutrition and Dietetics, Khoo Teck Puat Hospital, 90 Yishun Central, Singapore 768828, Singapore; yeong.hazel.el@ktph.com.sg; 4 Department of General Surgery, Tan Tock Seng Hospital, 11 Jalan Tan Tock Seng, Singapore 308433, Singapore; cheong_wei_huey@ttsh.com.sg; 5 Department of Geriatric Medicine, Changi General Hospital, 2 Simei St 3, Singapore 529889, Singapore; samuel.chew.t.h@singhealth.com.sg; 6 SingHealth Duke-NUS Medicine Academic Clinical Programme, 8 College Road, Singapore 169857, Singapore; 7 Yong Loo Lin School of Medicine, National University of Singapore, 10 Medical Drive, Singapore 117597, Singapore

**Keywords:** nutrition, malnutrition, discharge, Singapore

## Abstract

The nutritional status of hospitalised patients is often at risk or compromised and predisposed to further deterioration after discharge, leading to poor clinical outcomes, high healthcare costs, and poor quality of life. This paper aims to provide evidence-based best-practice recommendations to address this, supported by a national survey of healthcare professionals in Singapore and reviewed by a multidisciplinary expert panel under the Sarcopenia Interest Group of Society of Parenteral and Enteral Nutrition Singapore (SingSPEN). We advocate screening all patients with a validated tool which includes a disease activity/burden component, an easily accessible dietitian referral pathway for patients at risk of malnutrition, and an individualised nutrition care plan formulated and delivered using a multidisciplinary team approach for patients at risk or with malnutrition. A comprehensive team would include not only dietitians but also physicians, nurses, physiotherapists, speech therapists, and medical social workers working together towards a common goal. Information on why nutrition is important for good health and how it can be achieved should also be provided to all patients and their caregivers before and after hospital discharge. With the above recommendations, we seek to improve upon the current nutrition care processes at discharge for healthcare institutions in Singapore.

## 1. Introduction

Malnutrition among hospitalised patients is well documented, but its prevalence has not changed substantially since it was first reported in the 1970s [1,2,3]. Recent studies have reported a similar prevalence of malnutrition in hospitalised patients today to what there was then: 14.0–32.3% among surgical patients [4,5], 22.6–50.0% among patients with cancer [4,6,7,8,9], and 38.0–78.0% among ICU patients [10,11]. While national prevalence data in Singapore are limited, a recent scoping review of local studies reported a malnutrition prevalence of 14.7–65.0% in acute care settings [12]. Among hospitalised older patients (≥65 years) in Singapore, about 35.0% were malnourished at admission [13]. Malnutrition is associated with prolonged hospital stays, increased risk of postoperative complication, increased risk of re-admission, increased mortality, and higher healthcare costs [14,15,16]. Up to 20.0% of patients with cancer die from the consequences of malnutrition rather than the cancer itself [17]. Additionally, loss of muscle mass in malnourished individuals leads to functional decline [18], thereby increasing the risk of loss of independence.

Approximately 50.0% of hospitalised patients are malnourished at discharge [19]. Nutritional status often declines during hospitalisation [20,21,22,23], and it predicts a longer length of stay and hospital readmission within 6 months of discharge [24]. Weight loss during hospitalisation was associated with an increased likelihood of institutionalisation after discharge, and sarcopenia was associated with poorer functional status at 3 months after discharge [25]. Geriatric patients with a risk of malnutrition at discharge had a two-fold increase in mortality [26]. Those with sarcopenia and malnutrition/risk of malnutrition at discharge had a four-fold increase in mortality, compared with non-sarcopenic patients with normal nutrition [26]. Furthermore, patients may experience slow recovery of nutritional status after discharge, which may be prolonged in the elderly due to poor appetite, difficulty in chewing and swallowing food, and functional limitations that hinder meal preparation [27]. 

Given the negative impact of poor nutritional status on patients’ quality of life and health recovery, it is critical to address malnutrition during hospitalisation and prior to discharge by (1) identifying patients experiencing nutritional decline during hospitalisation and those who may be at risk of malnutrition at discharge, (2) treating malnutrition in these individuals during hospitalisation, and (3) formulating a nutrition care plan before discharge such that nutrition care can continue in the community. Currently, there is no local consensus to guide nutrition care practices after discharge in Singapore. Therefore, the aim of this paper is to provide evidence-based best-practice recommendations to ensure that patients’ nutrition status is addressed at and after discharge from the hospital.

## 2. Materials and Methods

These best-practice recommendations were developed by a multidisciplinary expert panel under the Sarcopenia Interest Group of the Society of Parenteral and Enteral Nutrition (Singapore; SingSPEN) comprising healthcare professionals (HCPs) in nutrition support, geriatrics, dietetics, and surgery. The research question we sought to answer was “What is the current best evidence to support best practices for nutrition care at and after discharge from hospital?”

Firstly, we sought the current best evidence from the literature, focusing on nutritional care after hospital discharge for best practices and existing gaps or barriers in this context, particularly in Singapore. A PubMed search was conducted with the following terms and their combinations: post-discharge, post-discharge planning, post-hospitalisation, nutrition, nutrition care, dietary care, nutritional intervention, malnutrition, sarcopenia, re-hospitalisation, nutritional care transition, dietitian, guidelines, consensus, Singapore, and Asia. We summarised the findings from the literature, which were used as a basis for these best-practice recommendations. Secondly, we conducted a short anonymous national survey to gain preliminary insights into existing practices and identify gaps, if any, in nutrition care after hospitalisation in Singapore (refer to Supplementary Materials for details on survey methodology and results). We received 242 responses, which included responses from doctors and dietitians from eight public general hospitals and four community hospitals (refer to Supplementary Materials for the list of hospitals). Finally, we convened to review the findings from the survey and formulate best-practice recommendations. The recommendations were refined through multiple iterations via two face-to-face meetings and e-mail correspondences.

## 3. Results

Aligned with existing guidelines and supported by data from the literature and the national survey (refer to Supplementary Materials), we developed these evidence-based best-practice recommendations as guidance to improve nutrition care after hospital discharge. The recommendations can be adapted for use by individual healthcare institutions in Singapore.

### 3.1. Nutrition Screening

Recommendation 1

*All patients should undergo nutrition screening within 24 h of admission.*

The Joint Commission International standard recommends nutrition screening within 24 h of admission and at frequent intervals throughout hospitalisation [28].

Recommendation 2

*Nutrition re-screening should be performed on a weekly basis during hospitalisation to identify individuals who may be experiencing nutritional decline.*

The NICE guidelines on nutrition support for adults recommend screening once a week for inpatients [29]. The recent ESPEN guidelines on hospital nutrition suggest re-evaluation of standard diet 5 days after hospitalisation [30]. Regular re-assessment of nutritional status is important for patients who have undergone surgery [31].

Recommendation 3

*If a patient is at risk of malnutrition, any HCPs regardless of profession should be able to make a direct referral to dietitians for further assessment and intervention.*

The EFFORT randomised controlled clinical trial demonstrated that compared with standard care, targeted nutritional intervention resulted in a 21% reduction in adverse outcomes and a 35% reduction in mortality in hospitalised adults at risk of malnutrition [32]. This underscores the need for all patients at risk of malnutrition to be referred to dietitians for assessment and intervention to minimise adverse outcomes. Furthermore, a recent umbrella review and meta-analysis concluded that nutritional intervention initiated in hospitals is effective at reducing mortality for up to 12 months in hospitalised adults with or at risk of malnutrition [33].

To allow prompt communication of nutrition screening results between nursing staff and other HCPs, the screening results should be included in the electronic health records [28]. The electronic healthcare system should be programmed to automatically initiate dietetic referral when a patient screens positive on the nutrition screening tool at admission and prior to discharge. This will ensure that all at-risk and malnourished patients can receive timely systematic assessment, appropriate and individualised interventions, and adequate monitoring and follow-up from a dietitian.

Additionally, we suggest conducting an audit of nutrition screening and referral process on a regular basis to facilitate consistent implementation.

Recommendation 4

*All patients should undergo nutrition screening at discharge.*

Patients experience worsening of nutritional status and weight loss during hospitalisation; this can be due to prolonged bed rest, illness-related anorexia, fasting for diagnostic procedures, treatment-related side effects (e.g., cancer treatment may induce nausea, vomiting, or diarrhoea), diseases that compromise the regular functions of the digestive system, and neglect of patients’ nutritional needs [23,34]. Nutritional decline during hospitalisation was associated with increased likelihood of re-admission and increased mortality after discharge [25,26,34,35]. Malnutrition remained prevalent in geriatric patients even up to 4 weeks after discharge [36]. Furthermore, nutritional recovery in older patients after hospitalisation is slow [37,38], and they may continue to have low nutritional intake and weight loss at home in the community [39]. 

It is therefore critical that nutrition screening is conducted before patients are discharged. However, data suggest that nutrition assessment at discharge is not performed on a regular basis by physicians [40]. Similarly, in Singapore, nutrition screening at discharge is not presently implemented in hospitals (Figure S1). We recommend that nutrition screening be performed on all patients prior to discharge to avoid the negative outcomes associated with unaddressed malnutrition.

Recommendation 5

*Use a validated screening tool that includes a disease activity/burden component.*

The use of a validated screening tool is recommended to identify patients at risk of malnutrition [41,42]. Several malnutrition screening tools have been evaluated and validated in various healthcare settings [43,44]. According to the Global Leadership Initiative on Malnutrition (GLIM), criteria for the diagnosis of malnutrition should include at least one phenotypic criterion (weight loss, low body mass index, or reduced muscle mass) and one etiologic criterion (reduced food intake or assimilation, or disease burden/inflammatory condition) [41]. Some validated tools, such as the Malnutrition Universal Screening Tool (MUST), the Mini Nutritional Assessment (MNA), and the Nutrition Risk Screening 2002 (NRS-2002), incorporate a disease activity/burden component [41,45]. Currently, various nutrition screening tools are used across institutions in Singapore (Figure S2). We encourage the use of the same screening tool by all public health institutions to facilitate better communication across healthcare institutions.

### 3.2. Development of an Individualised After-Discharge Nutrition Care Plan

Recommendation 6

*An individualised nutrition care plan should be formulated for patients who have been assessed to be malnourished or at risk of malnutrition during hospitalisation or at discharge.*

The presence of malnutrition or risk of malnutrition at admission suggests that the underlying issue is already present prior to hospitalisation [46], and therefore, appropriate discharge planning is critical before discharge to the community. An individualised nutrition care plan helps ensure that nutrition care continues after patients are discharged from the hospital to the community. Despite the importance of such a plan for patients, studies from Australia, Denmark, and the US reported that patients were often discharged without such a plan [47,48,49]. A survey among HCPs (including hospital, primary care, and community practices) conducted by the Canadian Malnutrition Task Force indicated that making and receiving a nutrition care plan post-hospital-discharge occurred infrequently [50]. Implementing nutrition screening at discharge and enabling automatic dietitian referral after screening, as required, would help ensure that dietitians are involved in discharge planning.

Recommendation 7

*The individualised nutrition care plan provided should include the following information:*

1. Target weight, achieving a body mass index (BMI) of at least 18.5 kg/m^2^ in those <70 years and 20 kg/m^2^ in those >70 years;
2. Target energy and protein intake;
3. Strategies to achieve target weight (i.e., food fortification, small frequent meals, nourishing fluids, and oral nutrition supplements [ONS]);
4. Duration of nutrition intervention;
5. Dietetics follow-up appointment;
6. Updates on nutrition care progress for primary-care physician.

The patient’s age, nutritional status, activity level, and medical conditions should be taken into consideration when determining their target weight, energy, and protein requirements. The GLIM consensus indicates a BMI < 18.5 kg/m^2^ for Asians < 70 years and <20 kg/m^2^ for Asians > 70 years as one of the criteria for the diagnosis of malnutrition [41]. In general, clinical guidelines recommend energy intake of at least 30 kcal/kg of actual body weight/day for older patients, those with an acute or chronic disease who are malnourished or at risk for malnutrition, or those with disease-related metabolic stress [30,51]. To achieve a weight gain of 0.5 kg/week, an additional energy intake of at least 500 kcal/day is needed [52,53]. Target protein intake of at least 1.2 g/kg of actual body weight/day is recommended for inpatients [30]. An intake of 1.2–1.5 g protein/kg of body weight/day may be required for older adults with acute or chronic disease, and up to 2.0 g protein/kg of body weight/day may be necessary for those with severe illness, injury, or severe malnutrition [54]. 

Food fortification refers to the addition of vitamins, minerals, energy, protein, or other nutrients, or a combination of them, to dietary intake to increase the energy and/or nutrient density [30]. The ESPEN guidelines recommend fortified food to support adequate dietary intake in older individuals with malnutrition or at risk of malnutrition [51], as evidence has shown that food fortification (through natural foods or specific nutrient preparations) was effective in improving intake with the same amounts of food [42]. Guidelines have also recommended small frequent meals—characterised by multiple small meal consumptions throughout the day—in patients with inadequate dietary intake, as evidence, albeit limited, supports its role in promoting higher energy and fluid intakes [55]. However, specific guidance on meal size, frequency, and timing may be required to prevent exacerbation of clinical conditions or other potential health complications [55]. Additional snacks and/or finger food are also recommended to facilitate dietary intake in older individuals with malnutrition or at risk of malnutrition [51].

ONS can improve intake of nutrients without reducing nutrient intake from food in older malnourished adults after discharge [56]. ONS is recommended for older adults with malnutrition or at risk of malnutrition [51], polymorbid medical inpatients with or at risk of malnutrition [57], patients who have undergone surgery [31], patients with cancer [17], patients in an intensive care unit [58], and older adults with malnutrition or at risk of malnutrition after discharge from the hospital to improve overall nutrient intake and body weight and lower the risk of functional decline [51]. The ESPEN guidelines also suggest that ONS offered to older adults provides at least 400 kcal/day, including 30 g or more protein/day, and that it should be continued for at least 1 month before re-assessment for effectiveness [51]. In hospitalised older adults aged 65 and above with two or more chronic diseases, nutritional support should continue for at least 2 months if they are at high risk or have established malnutrition in order to lower mortality and positively impact their clinical outcome [57]. The need for continual nutrition support following the intervention depends on dietitian review.

Nutritional interventions should be reviewed regularly by dietitians and continued until target dietary intake and target weight are achieved. Hence, timely follow-up is required to monitor the outcome of the interventions, re-assess nutritional status, and to re-adjust interventions if they are unsuccessful.

**Figure 1:**
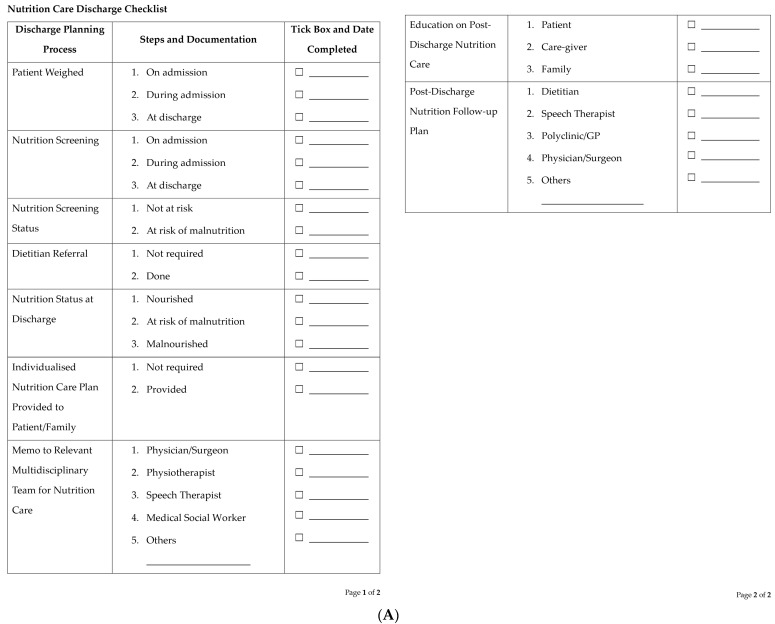

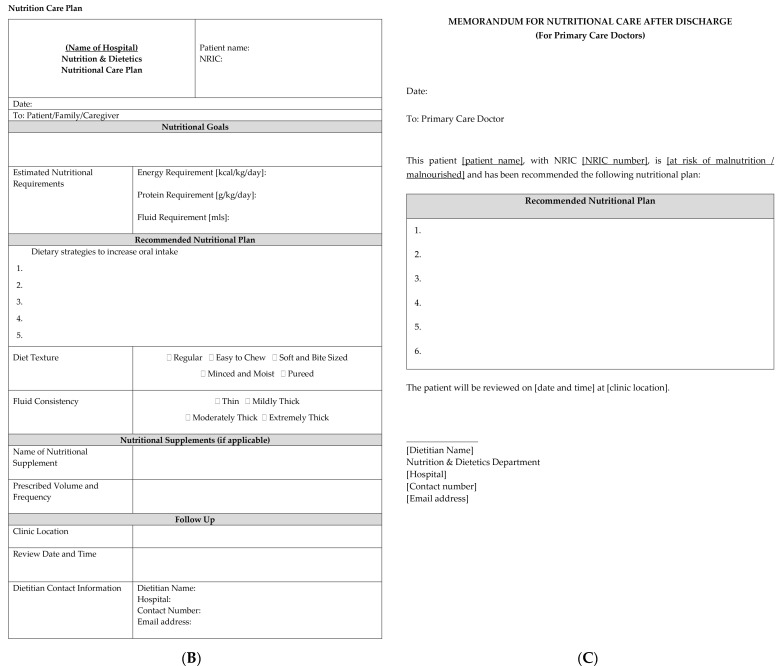
Proposed template for (**A**) a nutrition care discharge checklist, (**B**) a discharge nutrition care plan, and (**C**) a memo to primary-care physicians.

We propose a simple nutrition care discharge checklist and a discharge nutrition care plan template that can be individualised and provided to patients/caregivers prior to discharge (Figure 1A,B). This information should also be provided to primary-care physicians (Figure 1C) so they are aware of the nutrition care needs of the patients and can be involved in reinforcing nutrition care in the community.

### 3.3. Role of HCPs in the Planning and Delivery of After-Discharge Nutrition Care Plan

Recommendation 8

*Dietitians, physicians, nurses, physiotherapists, speech therapists, and medical social workers should collaborate in the planning and delivery of an after-discharge nutrition care plan.*

**Figure 2:**
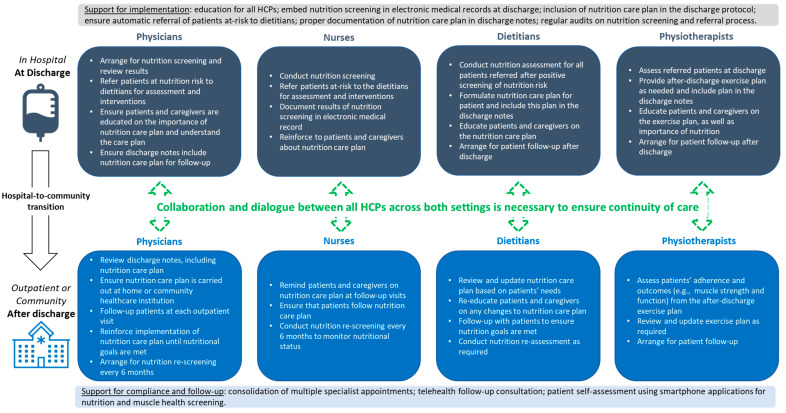
Roles of physicians, nurses, dietitians, and physiotherapists in planning, delivering, and implementing a nutrition care plan during hospital-to-community transition. Abbreviation: HCPs, healthcare professionals.

A collaborative and coordinated effort from relevant HCPs is required to ensure successful planning and delivery of an after-discharge nutrition care plan (Figure 2). Dietitians play a key role in the development of after-discharge nutrition care plans [50,59,60]. They are aware of all the available options related to the provision of nutritional support and follow-up in their institutions so that adequate arrangement can be made to ensure that nutrition care continues after patients are discharged from the hospital. Furthermore, dietitians play an important role in educating patients and their caregivers about the importance of the after-discharge nutrition care plan and in answering any questions or concerns. 

Physicians and surgeons provide support by emphasising the significance of nutrition as part of routine and essential clinical care to patients and their caregivers, and the importance of adhering to nutrition advice and after-discharge follow-up plans. Nurses further reinforce the importance of nutrition, ensure that patients and their caregivers understand the after-discharge nutrition care plan, and remind them about after-discharge follow-up plans. It is also important for primary-care physicians to be aware of the nutrition care needs of patients so they can ensure that patients continue to follow the nutritional care and follow-up plans.

The contribution of physiotherapists is often overlooked, but nutrition and physical therapy have synergistic effects in improving health and function [61]. Malnutrition affects muscle health; data from the SHIELD study show that four in five community-dwelling older adults in Singapore who were at risk of malnutrition had low muscle mass [62], and 76% were sarcopenic based on the Asian Working Group for Sarcopenia (AWGS) 2019 cut-offs [63]. Therefore, physiotherapists play an important role in educating patients on physical rehabilitation and exercises for bone and muscle health, which will lead to an overall improvement in appetite and function.

Speech therapists also play an important role in assessing swallowing problems that result in inadequate dietary intake [64]. They often work closely with dietitians to determine the appropriate diet consistency or texture that can be safely consumed by patients to optimise dietary intake [64,65]. Speech therapists also educate and train caregivers on how to feed patients optimally [66].

Medical social workers provide support to patients and their caregivers in terms of financial assistance and liaise with community care providers to facilitate a smooth transition from hospital to community. A recent retrospective study in a public tertiary hospital in Singapore demonstrated the importance of Medifund reimbursement for ONS and enteral tube feeds in improving the nutritional status of patients with financial difficulties [67]. Hence, the involvement of medical social workers is an essential component of after-discharge nutritional care.

### 3.4. Education on After-Discharge Nutrition Care

Recommendation 9

*Patients and their caregivers should be provided with adequate education related to after-discharge nutrition care.*

Educating patients and their caregivers on the importance of nutrition and exercise helps facilitate their active participation in improving their nutritional health during their hospital stay. Evidence from a recent systematic review suggests that nutritional support after hospital discharge may reduce mortality at 1 year after discharge by 37% and significantly increase nutritional intake and body weight in medical adult patients at risk of malnutrition [68]. The use of ONS after discharge also improved nutritional outcomes, skeletal muscle maintenance, and chemotherapy tolerance in patients at risk of malnutrition after gastric cancer surgery [69]. Findings from a study in Singapore suggest that an Ambulatory Nutrition Support model for after-discharge nutrition care which includes telephone calls and home visits in addition to outpatient clinics may address the challenge of low follow-up rates after discharge, leading to better nutritional status and outcomes [70].

If patients and their caregivers are not aware of the negative impact of malnutrition after discharge, they may not perceive nutrition as being important to their health (Figure S3). This may hamper uptake and compliance with nutritional interventions after discharge [27,71,72]. The lack of awareness of the importance of nutrition may also result in negative perception towards nutritional interventions, which in turn may cause patients and/or their caregivers to decline follow-up dietetic appointments (Figure S4).

It is therefore critical to ensure that patients and their caregivers understand the after-discharge care plan. They should also understand the importance of adhering to nutritional advice and the need to attend follow-up consultation. Simple, easy-to-understand written materials (e.g., brochures) or access to free resources (e.g., via mobile phone app or telehealth consultation) on nutrition should be made available to all patients and their caregivers at discharge to empower them to take charge of their own nutritional health and well-being. 

### 3.5. Collaborative Efforts Amongst Stakeholders to Support the Continuum of Care for Patients

Recommendation 10

*Collaborations amongst public health institutions, community healthcare partners, and community support groups are needed to support the continuum of care for patients.*

Proper discharge planning should support the recovery process of patients in the community, but the liaison between hospital, primary care, and community services are often lacking [50]. The hospital-to-community transition should incorporate dialogue and liaison between all key stakeholders (dietitians, physicians, nurses, physiotherapists, and medical social workers) between both setups. A qualitative study by the Dutch Malnutrition Steering Group suggests identifying a coordinator for nutrition care as an important way to improve collaboration and communication across healthcare settings [73].

There is currently no referral pathway in Singapore where discharged patients who are malnourished or at risk of malnutrition can continue to receive nutritional care and interventions. Structural support for the transition of nutrition care from hospital to community in Singapore is lacking. There is a demand for more dietitians to care for patients in the community (Figure S4).

## 4. Discussion

The recent literature has shown that a significant proportion of patients are malnourished on admission and at discharge [24,74,75]. Similarly, data from Singapore had reported longer hospital stays, higher hospital re-admissions after discharge and increased healthcare costs associated with malnourished patients [14]. There is an urgent need to take action to address this.

Based on the results of our national survey (Figure S3), the major reasons for patients declining after-discharge nutritional care include too many concurrent medical appointments to attend, transportation issues, the costs of care, the perception that nutrition is not important, perceived to be ineffective, the perception that the patient is too old to benefit, and the perception that the patient is not malnourished when they are.

Our best-practice recommendations aim to improve the nutrition care process at and after discharge by addressing these identified barriers to best care; we focus on nutrition screening at discharge, completion of an individualised after-discharge nutrition care plan, patient and caregiver education, as well as the need for a structured transition pathway from hospital to the community and/or primary-care setting. We further highlight the roles of HCPs as well as organisational support for successful planning, delivery, and implementation of the nutrition care plan (Figure 2).

We recognise that the implementation of these best-practice recommendations will depend on education and collaborative efforts among various stakeholders, as well as government policy in funding. It is imperative to improve the awareness and knowledge of HCPs, including primary-care physicians, on the importance of nutrition. The lack of knowledge or interest in nutrition care among non-dietetic HCPs is one of the most commonly reported barriers to nutrition care [46,48,50,76] and may also be the reason why nutrition care is not a priority at the point of discharge (Figure S4). HCPs should be encouraged to attend local courses on nutrition support. Medical education programmes for the HCPs should include practical aspects of nutritional health to ensure acquired knowledge and skills relevant to nutrition care can be implemented in routine clinical practice. Providing adequate nutrition education early during undergraduate medical training may inculcate the importance of nutrition care as an integral component in disease prevention and management [77].

In addition, efforts to highlight the importance of nutrition and its role in health and disease management must start in the community, as malnutrition often starts in the community. There is an urgent need to highlight the importance of good nutritional health for all age groups as part of the national public health messaging, not only to protect good health and function but also to support good muscle health and facilitate recovery from unexpected acute illness and trauma [32,78]. Public education should focus on promoting the fundamental concept that maintaining good nutritional health is essential to living well and strong, and proper nutrition and vitality are key to ensuring an independent and meaningful life across all ages.

There is also a need to ensure that improvements in the nutrition care process, once achieved, are sustained and spread to a new setting or unit [79]. This can be carried out through audits and feedback, recognition of good practice at the primary-care level, and identifying ‘champions’ in individual healthcare institutions who are dedicated to promoting and supporting the provision of best practices in nutritional care to patients [79].

Finally, to provide holistic and comprehensive nutritional care after discharge, a concerted effort by policy makers, healthcare institution leaders, and HCPs is necessary. This will include raising awareness, investing in education and training, and increasing the focus on collaboration during the transition of care from acute settings to the community/primary-care setting. We emphasise the importance of making malnutrition one of the key performance indicators for healthcare, as this can lead to improvements in both screening for and treatment of malnutrition in hospitalised patients and those in the community, as is evident from the efforts and experience of the Dutch Malnutrition Steering group [80]. There is a great need for the Ministry of Health to become one of the major stakeholders in promoting and advocating the importance of nutritional health for all patients.

The present paper has some limitations. The recommendations are based on a narrative review of current best available evidence with multidisciplinary expert-panel input and are not graded according to the GRADE system and should therefore be used accordingly. However, we believe that a narrative review can play an important role in highlighting the current clinical need and provide a starting point for discussions and further research amongst HCPs involved in after-discharge nutritional care. We agree that using a GRADE approach is important to empirically assess the evidence so that it can be more generalisable and used as guidelines without further assessment and considerations; we hope that our narrative review can play a role in the initiation of this process. Our survey results are based on a small number of responses and are limited to the Singapore population and HCPs. As such, the results may not be generalisable beyond the Asian context.

## 5. Conclusions

Data suggest that much remains to be done to address malnutrition during hospitalisation, at discharge, and after discharge. Our paper serves as the first step to improve the nutrition care process at discharge and after discharge, with the aspiration to foster changes in healthcare institutions across Singapore. Nutrition screening during hospitalisation and at discharge is essential to identify patients who are malnourished or at risk of malnutrition prior to discharge. Following which, an individualised nutrition care plan should be formulated and explained to these patients so they can return to the community, armed with knowledge and means to recover their nutrition status and overall health.

Almost 50 years after the first call to action, we now have the knowledge and tools to reduce and prevent malnutrition before, during, and after hospitalisation. What remains is for all of us to answer the call, with the will to make a difference.

## Data Availability

Data is contained within the article or supplementary material.

## Supplementary Materials

The following supporting information can be downloaded at: https://www.mdpi.com/article/10.3390/nu15214492/s1, Figure S1: Nutritional screening at admission, during hospitalisation and at discharge; Figure S2: Tools used for nutrition screening; Figure S3: Common reasons given by patients or caregivers for declining post-discharge nutritional care; Figure S4: Barriers to providing follow-up dietetic appointments following discharge.
