# Supplementary material for: Nutrition Care after Hospital Discharge in Singapore: Evidence-Based Best-Practice Recommendations
Source: Nutrients. 2023 Oct 24;15(21):4492. doi: 10.3390/nu15214492 (PMC10650134; doi:10.3390/nu15214492)
Supplement: Supplementary file 1 [file nutrients-15-04492-s001.zip › nutrients-2633771-supplementary.pdf]

Nutrition Care After Hospital Discharge in Singapore: Evidence-Based Best Practice Recommendations

Supplementary Information

To gain insights on existing practices of nutrition care after discharge, an anonymous online survey was developed and distributed via email by SingSPEN to all healthcare professionals (HCPs) practicing in Singapore public health institutions.

A total of 242 respondents from public health institutions (from eight general hospitals and four community hospitals) completed the survey; 26.4% are from Dietetics, 24.8% from Geriatric Medicine, 25.2% from other medical specialties (cardiology; emergency medicine; endocrinology; family medicine; gastroenterology and hepatology; internal medicine; intensive care medicine; neurology; oncology; ophthalmology; radiology; rehabilitation medicine; renal medicine; rheumatology), 14.5% from various surgical specialties (anesthesia; breast surgery; colorectal surgery; general surgery; hepato-pancreato-biliary surgery; obstetrics and gynecology; orthopedic surgery; surgical and orthopedic surgery; upper gastrointestinal and bariatric surgery), and 9.1% from nursing.

The survey responses were received from the following public health institutions:

No	Name of Hospital
1	Singapore General Hospital
2	Tan Tock Seng Hospital
3	Khoo Teck Puat Hospital
4	Changi General Hospital
5	National University Hospital
6	Ng Teng Fong General Hospital
7	Sengkang General Hospital
8	Alexandra Hospital
9	Ang Mo Kio Thye Hua Kwan Community Hospital
10	St Lukes Community Hospital
11	Yishun Community Hospital
12	Outram Community Hospital

Figure S1. Nutritional screening at admission, during hospitalisation and at discharge.

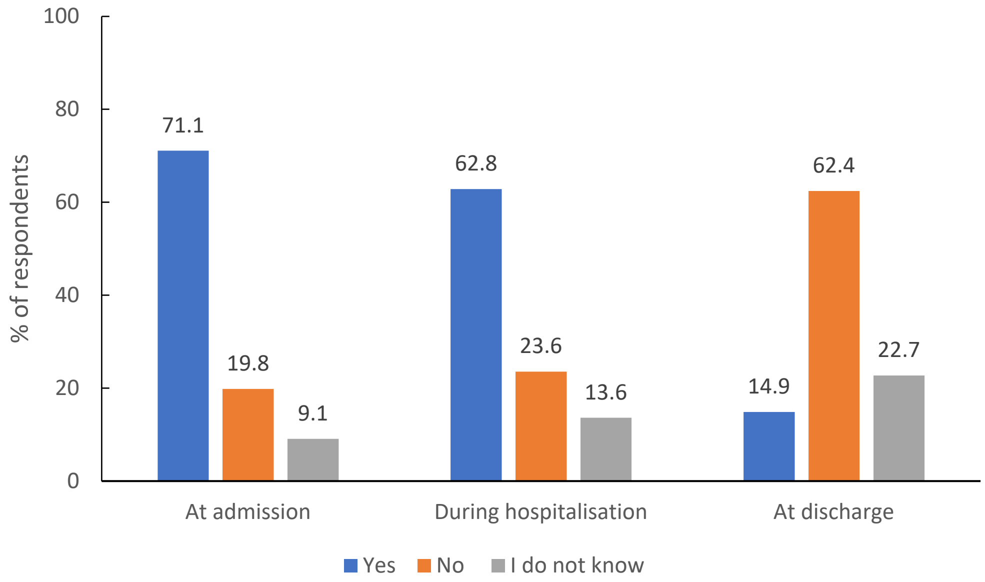

Figure S2. Tools used for nutrition screening.

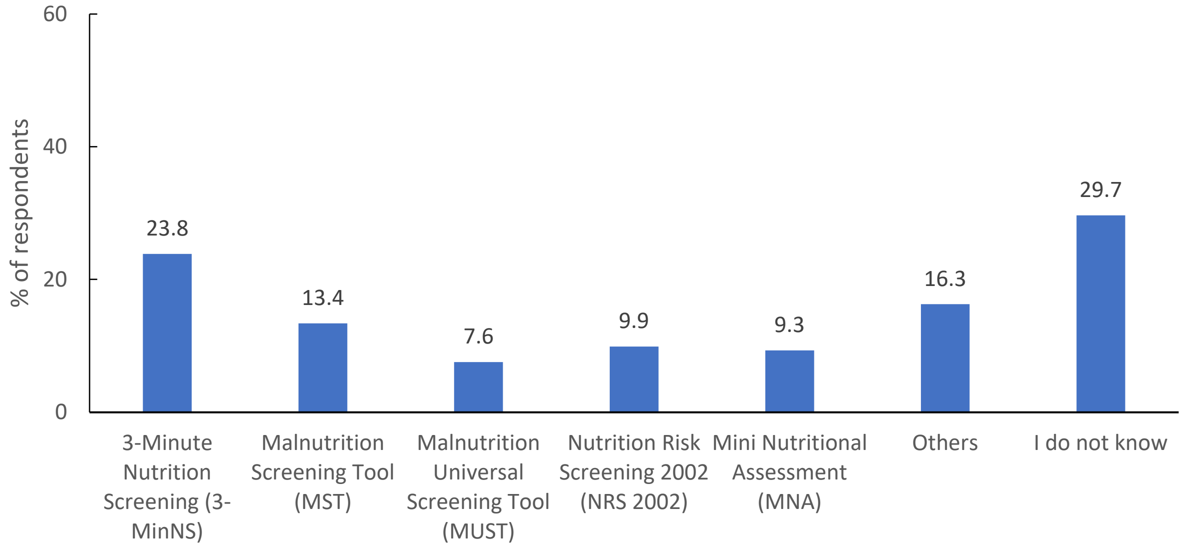

Respondents could choose more than one tool. Other tools/methods used for nutrition screening included hospital-specific in-house nutrition screening tool; clinical examination and history; integrated nursing records; Braden scale; subjective global assessment; and body mass index.

Figure S3. Common reasons given by patients or caregivers for declining post-discharge nutritional care.

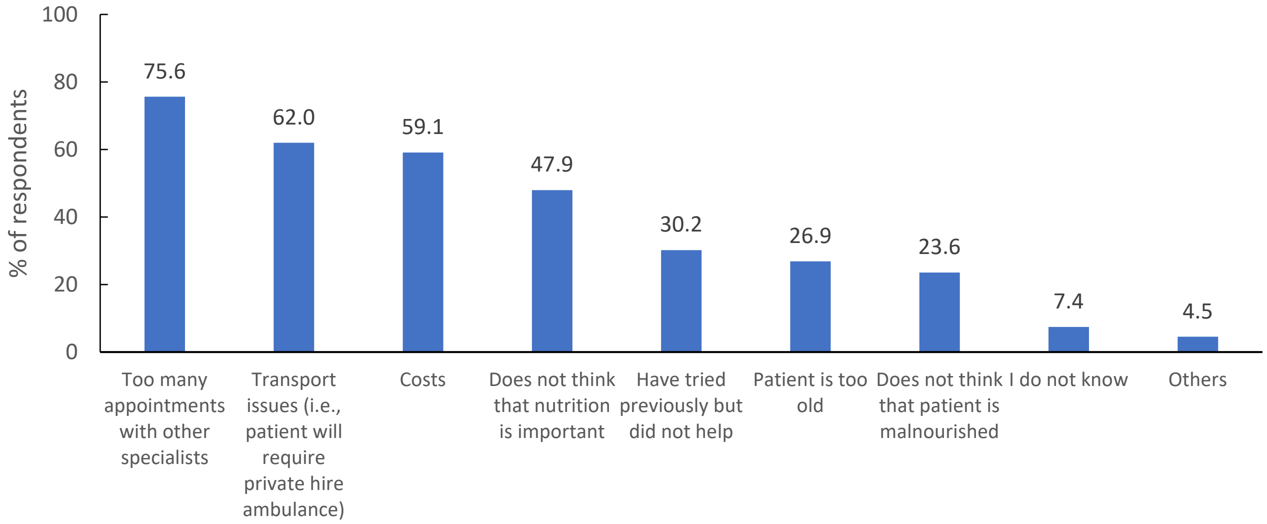

Respondents could choose more than one option. Other reasons reported included perception that malnutrition is normal for elderly / is a common aging process; patient and family feel they can manage on their own; poor appetite; and nutritional supplement products considered expensive or a waste of money.

Figure S4. Barriers to providing follow-up dietetic appointments following discharge.

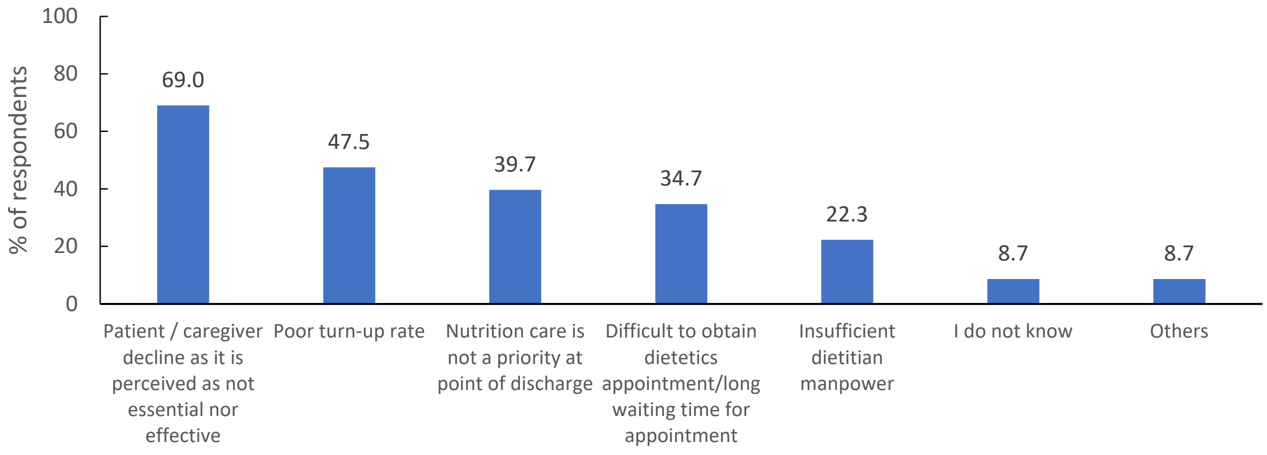

Respondents could choose more than one option. Other barriers reported included cost or absence of subsidy for post-discharge dietetic service; travel inconvenience for patients; dietitian follow-up not offered/referred by medical team; and unable to obtain same date as doctor's appointment.
